# Haematological Trends and Transfusion during Adult Extracorporeal Membrane Oxygenation: A Single Centre Study

**DOI:** 10.3390/jcm12072629

**Published:** 2023-03-31

**Authors:** Elliott T. Worku, April M. Win, Dinesh Parmar, Chris Anstey, Kiran Shekar

**Affiliations:** 1 Adult Intensive Care Services, The Prince Charles Hospital, Chermside, QLD 4032, Australia; 2 School of Medicine, University of Queensland, St Lucia, QLD 4072, Australia; 3 Intensive Care Unit, The Townsville Hospital, Townsville, QLD 4810, Australia; 4 Intensive Care Unit, Sunshine Coast University Hospital, Birtinya, QLD 4575, Australia; 5 Faculty of Medicine, Bond University, Gold Coast, QLD 4226, Australia

**Keywords:** extracorporeal membrane oxygenation, anticoagulation, packed red blood cells, restrictive transfusion

## Abstract

The temporal trends in haematological parameters and their associations with blood product transfusion requirements in patients supported with extracorporeal membrane oxygenation (ECMO) are poorly understood. We performed a retrospective data analysis to better understand the behaviour of haematological and coagulation parameters and their associations with transfusion requirements during ECMO. Methods: Patient demographics, haematological and coagulation parameters, plasma haemoglobin and fibrinogen concentrations, platelet count, the international normalised ratio (INR), the activated partial thromboplastin time (APTT), and blood product transfusion data from 138 patients who received ECMO in a single high-volume centre were analysed. Results: Ninety-two patients received venoarterial (VA) ECMO and 46 patients received venovenous (VV) ECMO. The median (IQR) duration of VA, and VV ECMO was 8 (5–13) days and 13 (8–23) days, respectively. There were significant reductions in haemoglobin, the platelet count, and the fibrinogen concentration upon initiation of ECMO. On average, over time, patients on VV ECMO had platelet counts 44 × 10^9^/L higher than those on VA ECMO (*p* ≤ 0.001). Fibrinogen and APTT did not vary significantly based on the mode of ECMO (*p* = 0.55 and *p* = 0.072, respectively). A platelet count < 50 × 10^9^/L or a fibrinogen level < 1.8 g/L was associated with 50% chance of PRBC transfusion, regardless of the ECMO type, and packed red blood cell (PRBC) transfusion was more common with VA ECMO. APTT was predictive of the transfusion requirement, and the decrement in APTT was discriminatory between VVECMO survivors and nonsurvivors. Conclusion: ECMO support is associated with reductions in haemoglobin, platelet count, and fibrinogen. Patients supported with VA ECMO are more likely to receive a PRBC transfusion compared to those on VV ECMO. Thrombocytopaenia, hypofibrinogenaemia, and anticoagulation effect the likelihood of requiring PRBC transfusion. Further research is needed to define optimal blood management during ECMO, including appropriate transfusion triggers and the anticoagulation intensity.

## 1. Introduction

Extracorporeal membrane oxygenation (ECMO) provides support for severe cardiac (veno-arterial, VA) and/or respiratory (veno-venous, VV) failure refractory to standard medical treatment [1,2,3,4]. Despite advances in the biocompatibility of modern ECMO circuits [5,6], and blood pumps [7,8], haemorrhagic and thrombotic complications remain significant and potentially concomitant complications of ECMO use [1,9,10,11,12]. There are several mechanical and biological mechanisms by which ECMO may adversely affect coagulation including, but not limited to, anticoagulation [13].

Critically ill patients who require ECMO frequently exhibit pre-existing coagulation dysfunction due to their underlying disease or treatment. ECMO may further exacerbate these alterations in coagulation and innate immune activation [14]; however, the independent significance is unclear. The platelet is an important immune mediator [14,15,16], that is pivotal in the cell-based model of coagulation [17]. Both quantitative and qualitative platelet defects leading to transfusion are common during ECMO [18,19]. Dynamic alterations in coagulation during ECMO may last days to several weeks and place the patient at a variable risk of haemorrhage and thrombosis. Exposure to nonendothelial surfaces is proinflammatory [20], and deleterious to the endothelial glycocalyx [21], whilst turbulent flow, high shear stress, and stasis in blood pumps may directly incite blood trauma [14,22,23]. Contemporary centrifugal pump designs [7,8], and heparin bonding of circuits [5] have significantly improved the biocompatibility of modern ECMO [24]. Nonpulsatile flow during ECMO can also produce acquired von Willebrand disease [1,25,26,27], and unfractionated heparin use may be complicated by the development of HIT (heparin induced thrombocytopaenia). While an adequate haemoglobin concentration is key to ensuring the oxygen carrying capacity is optimal, the potential harm associated with blood product transfusion is well recognised. Transfusion of multiple blood products may produce dilutional coagulopathy and add significantly to the cost of care [4], Additional risks include transfusion-related circulatory overload, acute lung injury, and transfusion-related immunomodulation [28], which may further impair organ function recovery [29]. It has previously been shown that greater red cell utilisation is associated with mortality in ECMO-treated patients [9,30,31]. In this retrospective study, we investigated the temporal trends in the plasma haemoglobin (Hb) and fibrinogen concentrations, platelet counts, international normalised ratio (INR), activated partial thromboplastin time (APTT), and blood product transfusion. Data were collected and analysed from patients supported with ECMO.

## 2. Materials and Methods

We conducted a retrospective, descriptive study of all patients who received either VA or VV ECMO at a single centre from April 2009 to December 2016. Patients for whom ECMO circuitry was used to provide temporary mechanical circulatory support in a uni or biventricular assist device configuration were excluded. ECMO was provided by the ROTAFLOW^®^ or CARDIOHELP^®^ systems (Gettinge, Germany). Anticoagulation was achieved with unfractionated heparin, and dosing was guided by regular APTT measurements. Local protocols recommend the maintenance of an APTT of between 50 and 60 s.

Demographic, laboratory, and transfusion data from 138 patients who received ECMO were reviewed. Demographic data were collected prospectively by the Data Management Unit. Laboratory data and transfusion data were collected from an online pathology system. Values were ascertained from pre-ECMO (collected before the initiation of ECMO) to the last day of ECMO for each individual patient. Day 0 is the day of initiation of ECMO. For haemoglobin, platelets, and fibrinogen, the nadir over a 24-h period was taken, and for APTT and INR, the peak value over the same time period was collected. Fibrinogen levels of less than 1.7 g/L were measured as clottable fibrinogen, and higher levels were derived. The transfusion data were collected based on the units of packed red blood cells (PRBC), platelets, and cryoprecipitate issued by the blood bank for individual patients.

Ethics approval was obtained from the local Research, Ethics, and Governance Unit (HREC/17/QPCH/152).

### Statistical Analysis

The statistical software package Stata^®^ Version 15.0, (Stata Corporation, College Station, TX, USA) was used. For demographic data, normal data were summarised as the means (SD) and analysed using a two-tailed non-paired *t*-test. The Shapiro–Wilk test was used to identify normally distributed data. Non-normal data were summarised as medians (IQR) and analysed using the Wilcoxon rank-sum test. Proportionate data were summarised as the number (%) and analysed using the Fisher’s exact test. All trend graphs plot the mean parameter over the patient group for each ECMO mode. Transfusion types are plotted as the mean unit per day. Lines of best fit are included from Day 0; however, baseline pre-ECMO data were excluded from the trend analysis. As the data become sparse after Day 24, confidence intervals cannot be relied upon beyond this point. Where appropriate, the cross-sectional time series data were analysed to Day 60 using univariate linear regression. Data were analysed to Day 60. The β value is the slope of the linear regression line—a negative value denotes a fall in the value of the variable over time. The *p* value for the slope indicates whether the regression slope is significantly different from zero (i.e., no association over time). In the time-based comparisons between VA and VV ECMO, including product transfusion, separate statistical models that included a term for the mode of ECMO for each haematological variable were constructed. As before, these compared the change in the variables over time with the extra mode term included. The INR/APTT versus time and transfusion requirement results are represented as the median (IQR) and univariate analyses. The analysis time was from Day 0 and was limited to 28 days due to scarcity of data beyond this time.

## 3. Results

### 3.1. Demographics

A total of 138 patients were included in this study. Ninety-two patients received VA ECMO, and 46 patients received VV ECMO (Table 1). The median (IQR) duration spent on VA ECMO was 8 (5, 13) days, and for VV ECMO, it was 13 (8, 23) days. Every patient included in the study received blood product transfusion. In comparison with patients on VV ECMO, patients who received VA ECMO were older, predominantly male, had a higher mortality rate, and had a lower ECMO duration and ICU length of stay. (Table 1). Patient indication classifications broadly align with those presented by the registry data for Australia and New Zealand Extracorporeal membrane oxygenation–EXCEL [32]. The majority of VVECMO was utilized for patients with the respiratory virus ARDS or following lung transplant, while the majority of VAECMO cases were patients with postcardiotomy and nonmyocardial-infarct-related cardiomyopathies. Further differentiation by diagnosis is provided in the supplementary tables. Sepsis as an admission diagnosis is not able to be further detailed in retrospect by aetiology (e.g., pulmonary vs. nonpulmonary sepsis).

VA ECMO was primarily received by patients with nonmyocardial infarction and noncardiotomy-related cardiomyopathies, whereas VV ECMO was mostly used for acute respiratory distress syndrome. There was no statistically significant difference in the use of either mode for sepsis (Table 2).

### 3.2. Haematological Parameters

A comparison of the haemoglobin concentration, platelet count, and fibrinogen concentration between the pre-ECMO status and Day 0 revealed highly significant reductions in all three variables. The mean pre-ECMO haemoglobin concentration was lower, and the mean pre-ECMO fibrinogen level was higher in the VV ECMO group (Table 3).

**Figure 1:**
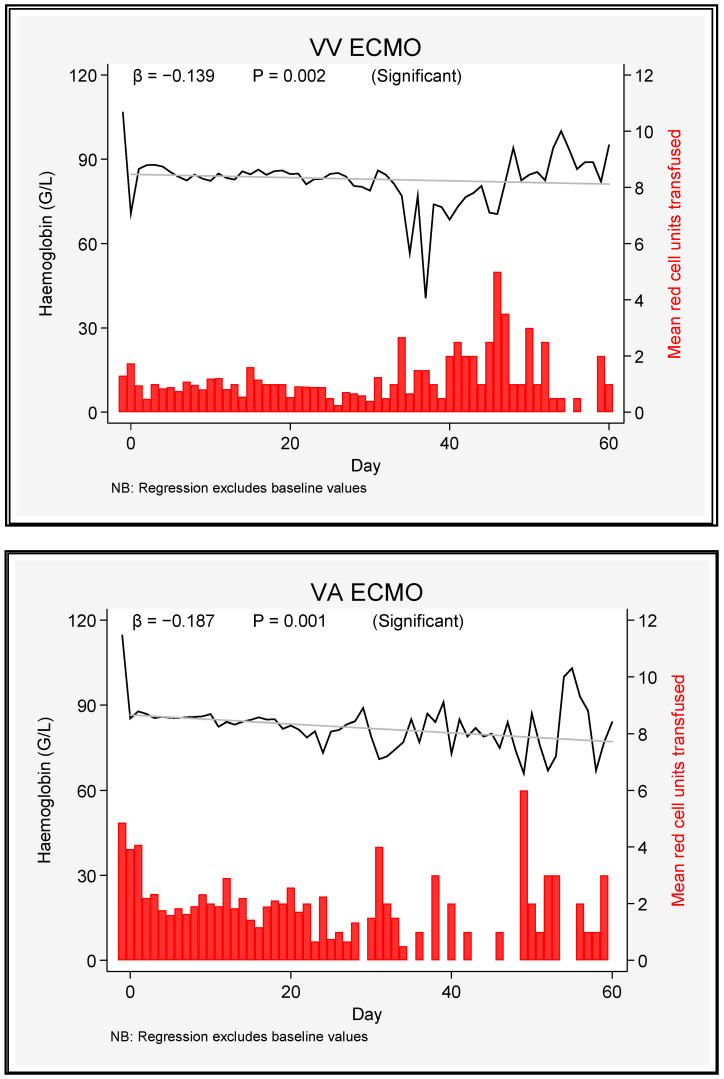
Haemoglobin level and packed red blood cell transfusion requirements in VV and VA ECMO patients over 60 days.

There was no difference in either the absolute level of haemoglobin or its trend between the two modes of ECMO. Haemoglobin levels decreased slowly but significantly over time (Figure 1).

### 3.3. Transfusion Requirements

**Figure 2:**
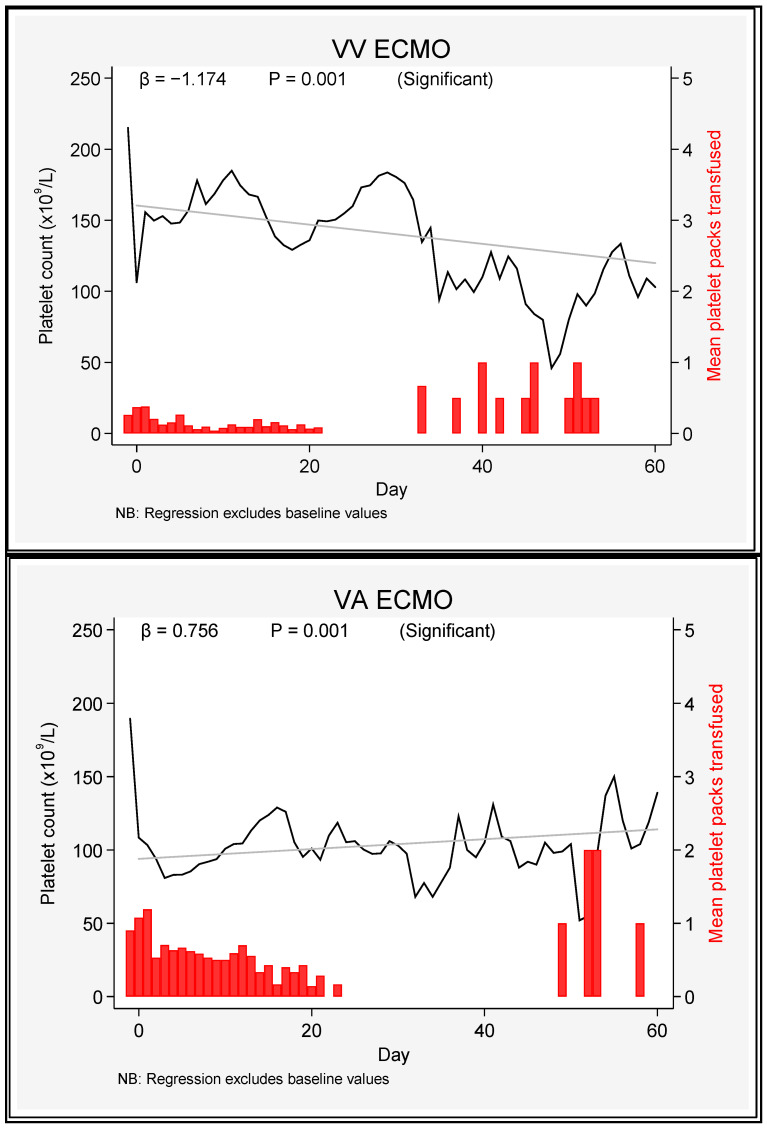
Platelet count and platelet transfusion requirements in VA and VV ECMO patients over 60 days.

On average, over time, patients on VV ECMO had platelet counts 44 × 10^9^/L higher than those on VA ECMO (β = +44, *p* ≤ 0.001) (Figure 2).

**Figure 3:**
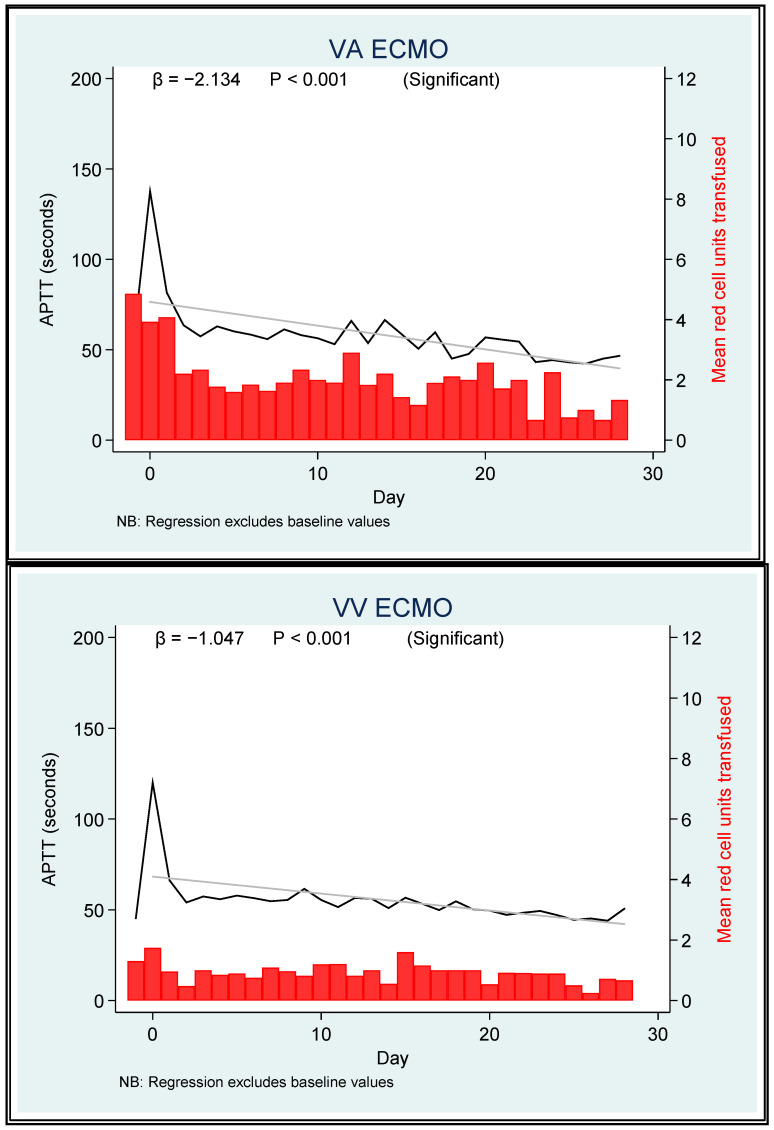
APTT and PRBC transfusion requirements in VA and VV ECMO patients over 28 days.

For fibrinogen and APTT, the ECMO mode was not a significant predictor (*p* = 0.55 and *p* = 0.072, respectively) (Figure 3). 

**Figure 4:**
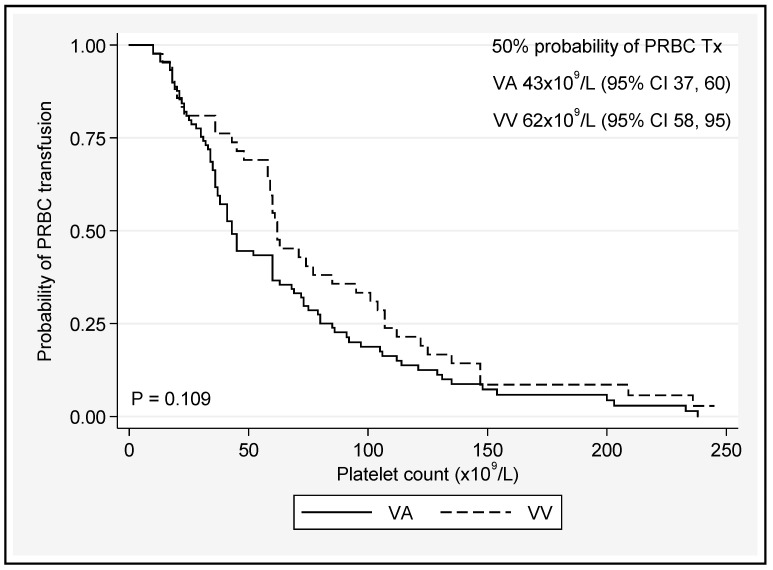
Platelet count cut-off value associated with PRBC transfusion in VA and VV ECMO patients. Tx = transfusion.

**Figure 5:**
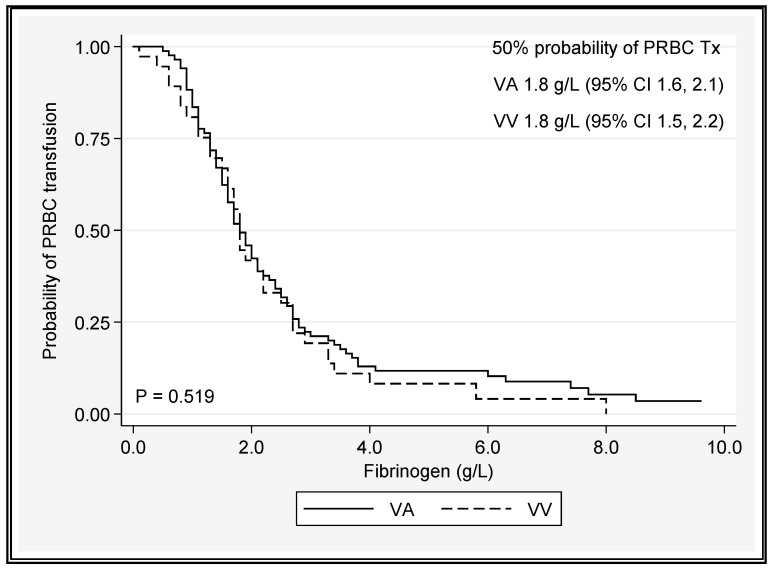
Fibrinogen level cut-off value associated with PRBC transfusion in VA and VV ECMO patients. Tx = transfusion.

A platelet count < 50 × 10^9^/L or a fibrinogen level < 1.8 g/L was associated with 50% chance of PRBC transfusion, regardless of the ECMO type (Figure 4 and Figure 5 and Supplementary Figure S1).

**Figure 6:**
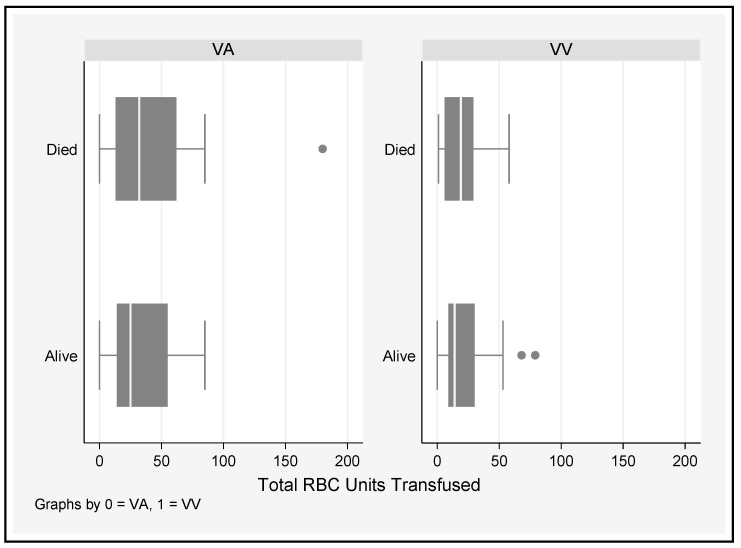
Comparison of total PRBC units tranfused between survivors and nonsurvivors who underwent VA and VV ECMO.

A cut-off value of 50% was chosen, as this is a common cut-off point in time-to-event analyses. There was no significant difference in the total number of PRBC units transfused in survivors and nonsurvivors who underwent either VA or VV ECMO (Figure 6).

In both the platelet and fibrinogen analyses, no significant difference between the ECMO modes was identified when predicting red cell transfusion. The intermediate probabilities of transfusion can be inferred from the Kaplan–Meier plots. When PRBC transfusion requirements were plotted against INR and APTT versus time, the most useful predictor of the need for PRBC transfusion was APTT. A drop in APTT of 1.2 to 1.8 s per day over 28 days was associated with survival, irrespective of the ECMO mode (predicted drop from 69 s (Day 1) to 36 s (Day 28) in VV survivors; predicted drop from 76 s (Day 1) to 27 seconds (Day 28) in VA survivors).

A fall of 2.5 s per day over 28 days was associated with mortality in the VA group. There was a drop from 98 s (Day 1) to 28 s (Day 28) in VA nonsurvivors (Table 4).

The relationships of the number of PRBC units transfused with the independent predictors of APTT, outcome, ECMO mode, and time were examined in a cross-sectional time series mixed effect univariate linear regression model. The significant positive predictors of red cell transfusion between Day 1 and Day 28 were an increasing APTT (*p* < 0.001) and the VA ECMO mode (*p* = 0.005). Overall, 3262 units of PRBC were transfused during the study period (Table 5). 

Patients on VA ECMO received greater transfusion volumes than those on VV ECMO across all products, even when adjusted for respective ECMO days.

## 4. Discussion

In this study we evaluated trends in the haemoglobin concentration, platelet count, and fibrinogen concentration during ECMO. There were significant reductions in haemoglobin, platelets, and fibrinogen from pre-ECMO levels to the day of ECMO initiation in both ECMO types but no pathognomonic temporal trend by ECMO mode during the 60-day observation period. This finding is similar to previously published data [4]. Mortality was 48.9% in the VAECMO cohort, substantially higher than that experienced by patients undergoing VVECMO (17.4%; *p* < 0.001). The former group were predominantly supported for cardiomyopathies of nonischaemic origin or postcardiotomy/post-transplant, whereas VVECMO was deployed for ARDS in the majority of cases. Our VVECMO survival data compare well with the ELSO registry data from 2017–2021 (VVECMO mortality 42%) [33]. In the Australia and New Zealand Extracorporeal membrane oxygenation registry (EXCEL) report from 2019–2021, the risk-adjusted mortality for VVECMO was 29%, while that of VAECMO was 45% [32].

Our VVECMO survival is excellent and is testament to the unit experience gained during the H1N1 pandemic, robust protocols surrounding ventilation management, and strict selection criteria for extracorporeal support. Our VAECMO mortality rate, however, may be higher than that of other programs due to a relatively large proportion of our cohort receiving postcardiotomy support (25%), which is traditionally associated with high mortality rates nearing 70% [34,35].

Furthermore, a low proportion of patients at our centre were treated for myocardial-infarct-related cardiogenic shock (4.3%—Table 2). This group may have excellent outcomes with early mechanical support and early revascularization therapies, whereas the majority of our ECMO recipients are typically referred through the statewide chronic heart failure service administered by our hospital, and thus there are larger numbers of patients with decompensated chronic heart failure and inflammatory cardiomyopathies. Our VAECMO mortality is thus in reasonable agreement with outcomes documented in other registries reporting the experiences of patients of similar demographics [36].

On average, over time, patients on VV ECMO had platelet counts 44 × 10^9^/L higher than those on VA ECMO. Fibrinogen and APTT did not vary significantly based on the mode of ECMO. A platelet count < 50 × 10^9^/L or a fibrinogen level < 1.8 g/L was associated with 50% chance of PRBC transfusion, regardless of the ECMO type, and transfusion was more common during VA ECMO support [37]. An increasing trend in APTT (*p* < 0.001) was associated with greater likelihood for transfusion between D1 and D28, as was VAECMO therapy (*p* = 0.005) (Table 4 and Table 5). In many studies, bleeding remains the most common and serious complication for patients on ECMO [9,31,38,39]. Aubron and colleagues [9] assessed haemorrhagic events by the total amount of PRBC transfused during ECMO and found an association between volume and hospital mortality in both VA and VV ECMO. The current study could not elicit a significant difference in the total number of units of PRBC transfused between survivors and nonsurvivors; however, this association has been well documented [30,40]. Even small transfusion volumes may be associated with increased morbidity and resource consumption along a continuum of risk [30,41], so defining appropriate thresholds for use in ECMO patients is vital to reducing the cost and improving outcomes of care. The extracorporeal life support organisation (ELSO) suggests platelet transfusions to maintain a platelet count of >80,000 cells/mm^3^ and the maintenance of normal fibrinogen levels (2.5–3 g/L) [42]; however, recent expert consensus suggests that a more restrictive transfusion policy [43,44] in nonbleeding patients may be entertained (platelets > 50,000 cells/mm^3^, Fibrinogen ≥ 1 g/L) [44], corroborating the threshold values for platelet counts observed in this current study. It should be noted that the ELSO recommendations are not directed at minimising the risk of product transfusion, but rather to limiting the incidence intracranial injury and optimizing oxygen delivery. There was a demonstrable drop in haemoglobin from pre-ECMO values immediately after the initiation (day 0) of ECMO and then a gradual decline over time, regardless of the ECMO configuration. The reduction in haemoglobin during ECMO is likely multifactorial: bleeding, haemolysis, haemodilution, and possible sequestration of RBCs in the ECMO circuit [4].

Our service utilises two platforms for ECMO support that we prime exclusively with normal saline: the ROTAFLOW^®^ centrifugal pump (Getinge, Germany) coupled with a PLS-I oxygenator (total priming volume of 656 mL and the Cardiohelp^®^ (Maquet, Rastatt, Germany) combined with a HLS Set Advanced 5.0 (total priming volume 570 mL). Early patients would exclusively have been treated with the ROTAFLOW^®^ device, while there has been a transition towards the predominant use of the Cardiohelp^®^ device in more recent years. As an institution, we anecdotally run patients ‘dry’ with negative fluid balances targeted in patients with cardiogenic shock, particularly post-transplant/postcardiotomy and in patients with ARDS. At the time of ECMO initiation, it is not uncommon for patients to require intravenous volume resuscitation of ~1 L in order to establish flows, which may further add to the degree of haemodilution. These data could not be analysed for the purposes of this retrospective study, and the recording of fluid administration in patients cannulated to ECMO in the operating theatre is similarly unreliable.

Clinicians generally targeted a haemoglobin concentration of 9–10 g/dL during this study. In an international multicentre survey, services with >24 ECMO runs per year transfused at a haemoglobin concentration of 8.4 (7.7–8.9) g/dL vs. 9.6 (9.1–10.0) g/dL in ECMO centres performing <12 runs per annum [45]. Outside of ECMO, multiple studies conducted in critically ill cohorts have supported restrictive transfusion policies [46,47,48], and it has been shown that DO_2_/VO_2_ relationships may not be positively influenced by transfusion outside of extreme anaemia [49,50].

In an observational study of 45 paediatric ECMO patients, the majority of transfusions occurred at SvO_2_ ≥ 70%, and less than 10% of packed red cell transfusions were associated with significant increases in SvO_2_ or cerebral saturations inferred by NIRS [51].

Prospective studies of transfusion strategies for ECMO patients are somewhat lacking, with variable practices encountered [31,45,52,53,54]; however, retrospective observational data suggest that a restrictive threshold of <80 g/L may be safe and provide substantial cost savings [55]. In a prospective international multicentre observational study of 604 patients receiving VVECMO, the mean pretransfusion threshold was 8.1 g/dL, and 83% of patients received at least one unit of blood during ECMO [56]. Transfusion was associated with lower risk of death only when the Hb was <7 g/dL [HR 0.15 (0.03–0.74)]. Swol et al showed that mortality was increased [RR 1.73 (1.134–2.639)] in patients transfused with a haematocrit > 31% [57]. A recent systematic review and meta-analysis of both VV and VAECMO supports a restrictive transfusion practice, which may reduce mortality and AKI without significantly prolonging the ECMO run [52]. Agerstrand et al employed restrictive transfusion (trigger < 7 g/dL), low intensity anticoagulation (APTT 40–60 s), and autotransfusion of blood from the circuit after decannulation [40]. This bundle of care substantially reduced levels of transfusion. Cahill et al demonstrated a 45.4% reduction in packed red cell transfusions, and a 62.9% reduction in platelet transfusion in postcardiotomy ECMO patients after adopting their own restrictive protocol, translating to large cost benefits [58]. The venoarterial [37] and postcardiotomy groups generally experience the greatest transfusional burden [30], with a large systematic review of 4000 patients revealing a median of 3.86 (2.51–5.22) units of packed red cells transfused per day of VAECMO vs. 1.23 (0.89–1.57) *p* < 0.001 in VVECMO patients [37]. Blood conservation requires not only tolerance of more restrictive thresholds, but meticuluous anticoagulation, autologous transfusion [40,59] at the time of decannulation, and proactive surgical management of bleeding. Multidisciplinary blood management [60] should also include the prioritization of percutaneous over open surgical ECMO cannulation, although this has implications for training and ECMO provision at largely surgically led services [37,61]. In our study, the observed decrease in platelets after the initiation of ECMO is likely to be multifactorial and related to platelet activation, consumption by the ECMO circuit, and bleeding [4,62]. Haemolysis and shear blood trauma may further add to the transfusion burden. Mechanical left ventricular unloading strategies are a confounder that is unique to VAECMO, which may add to haemolysis and bleeding risk and thus partly explain the excess number of transfusions in VAECMO patients with comparison to VVECMO patients [63]. Acute kidney injury is also more common in VAECMO and is often present at the time of cannulation [64], with prognostic relevance [65]. Renal replacement therapy is employed in 20–100% of these patients [66], thus exposing them to further potential for mechanical blood trauma. In our study, 39.1% (18/46) of VVECMO patients and 50% (46/92) of VAECMO patients underwent renal replacement therapy during ECMO. Plasma-free hemoglobin values were not collected during the study period; however, just 2/92 VAECMO patients received mechanical unloading strategies: n = 1 surgical left ventricular venting cannula and n = 1 intra-aortic balloon pump. Such low numbers do not permit further analysis. No percutaneous left ventricular assist devices, such as the Impella^®^, which are frequently associated with bleeding and haemolysis [63,67,68], were implanted in any patients.

The relationships among APTT, bleeding, and ECMO survival are complex. A reductions in APTT over time appeared to correlate with survival in VVECMO patients but was less discriminatory in VAECMO patients (Table 4). Increasing APTT values were also predictive of red cell transfusion. Of course, reduced APTT values may correspond to a number of clinical situations, each with differing implications on outcomes. For example, APTT measurements may simply fail to accurately reflect the level of anticoagulation during ECMO, and for this reason, AntiXa levels are increasingly relied upon instead [69,70,71,72,73]. The APTT may also be reduced in the setting of heparin resistance due to an antithrombin III (ATIII) deficiency. ATIII is a plasma alpha 2 glycoprotein whose anticoagulant action is increased 1000-fold in the presence of heparin [74]. ATIII levels are commonly lower during ECMO [54] and may also be reduced by attendant haemodilution [75]. Replacement of ATIII (via concentrate or larger volumes of fresh frozen plasma) during ECMO has thus been proposed to improve heparin sensitivity, but replacement remains controversial, as this practice has not been associated with a reduction of thrombotic sequalae or improved circuit longevity and may be associated with increased rates of bleeding and need for factor/platelet transfusion [76]. Our local practice is to not measure ATIII levels, as we do not replace them in case of a deficiency.

Lastly lower APTT levels may be intentional, or necessary. Given the high proportion of VAECMO patients receiving postcardiotomy ECMO, low APTT values may reflect contraindications to conventional intensity anticoagulation due to surgical bleeding. Likewise, a trend towards a reduction in APTT would be anticipated in patients who have been liberated from ECMO and have no enduring indication for therapeutic anticoagulation beyond mechanical support. In this retrospective setting, it is unwise to imply causality without further contextual information.

The initial platelet count was lower in the VA ECMO group compared to the VV ECMO group, which may reflect the significant use of VAECMO in a postcardiotomy setting at our centre where antiplatelet therapy and cardiopulmonary bypass are frequent co-exposures. In contrast, sepsis was diagnosed in a greater proportion of patients on VV ECMO (although not statistically significant). Fibrinogen levels decreased over time with both modes of ECMO, but attrition was more rapid during VV ECMO. This reduction may be due to inflammatory cascade activation and the adsorption of fibrinogen onto the ECMO circuitry [77]. As fibrinogen was not measured daily in our ECMO patients, there were insufficient data to determine the trend evolution of this factor over time; however volumes, of cryoprecipitate transfusion are described. Unsurprisingly, higher volumes of cryoprecipitate were administered to VAECMO patients vs. VVECMO patients. Our local practice during the study period was to utilize cryoprecipitate alone for fibrinogen deficiency; fibrinogen concentrate was not locally available until 2018, and other factor concentrates e.g., prothrombin complex concentrate, were not administered to ECMO patients as per the unit policy.

The platelet nadir during ECMO has previously been associated with PRBC transfusion [31], and in the current study, a platelet count of <50 cells/mm^3^ and a fibrinogen concentration of <1.8 g/L increased the probability of PRBC transfusion by 50%. There was also a trend towards transfusion at a platelet count of 43,000 cells/mm^3^ on VAECMO and 62,000 cells/mm^3^ on VVECMO, although this difference did not reach significance. In this study, we were not able to show that the requirement for PRBC transfusion was due to excess bleeding, similar to previous reports [58]. There are emerging and important differences in coagulation status between VAECMO and VVECMO patients which may have implications for personalized anticoagulation approaches by modality [78]. VA ECMO may be associated with a loss of normal cardiac pulsatility, which can produce excess inflammation [20] and acquired Von Willebrand syndrome [27,79]. Furthermore, reductions in transpulmonary blood flow during peripheral VAECMO may limit the breakdown of bradykinin [62] and thus potentiate the contact pathway of coagulation [80]. Aubron et al. found VA ECMO to be associated with increased blood product transfusion compared to VV ECMO. Nonsurvivors of ECMO (both VA and VV) also received greater volumes of blood and platelets, whereas in the multivariate analysis, the volume of RBC units transfused was associated with mortality in VA ECMO alone [9]. The strengths of the current study include the large populations of both VA and VV ECMO patients with consistent data collection methods adding to the repository of knowledge around this important topic.

However, our study has some important limitations, including the monocentric data collected over a long period during which transfusion attitudes, the ECMO case volume, and anticoagulation monitoring practices evolved. There is a lack of data concerning haemorrahgic and thrombotic sequalae impacting patient management, e.g., oxygenator thrombosis which would allow more robust interpretation of APTT and transfusion data. Additionally, local anticoagulation and blood management policies may not be generalisable to other noncardiothoracic surgical centres. In this retrospective study, we were unable to examine whether transfusion occurred in response to a specified haemoglobin trigger, a perceived requirement for a higher oxygen-carrying capacity, or to active haemorrhage. The role of cannula type, ECMO device, and varying pump speeds was not interrogated in this study, shear stress and the haemolytic and thrombotic potential were influenced by the pump design and operation speed [23,26,27,81,82,83,84].

We did not analyse the effect of the transfusion volume by year of patient inclusion, which may have been influenced by the earlier reliance on ROTAFLOW^®^ and the more recent transition to Cardiohelp^®^ platforms as well as the increasing practice volume. A lack of granular data regarding ECMO indications is also relevant, for example, early in the course of data collected (2009) a large proportion of VVECMO recipients were H1N1 Influenza-related, and in the Australasian experience, they were frequently young with a relatively low mortality rate with respect to bacterial-pneumonia-related ARDS, for example [85].

The cannula type and mode of insertion (percutaneous vs. hybrid or cut-down) are not reported, but likely differed between patients cannulated in the ICU (routinely percutaneous and physician led) and those cannulated in the operating theatre by surgeons which may have further influenced the bleeding risk and response [61]. The association of APTT reduction during ECMO with survival (Figure 3) suggests low-intensity anticoagulation protocols [86,87], and even anticoagulant-free ECMO [88] merit ongoing prospective evaluation, as does the use of antiXa monitoring; however, without thrombotic event documentation, the association identified in the current study must be received with caution. 

## 5. Conclusions

ECMO support was associated with reductions in haemoglobin, the platelet count, and fibrinogen. Patients supported with VA ECMO were more likely to receive a PRBC transfusion compared to those on VV ECMO. A platelet count < 50 × 10^9^/L or a fibrinogen level < 1.8 g/L increased the likelihood of PRBC transfusion. Reductions in APTT were associated with reduced risks of transfusion and mortality. Further research is needed to define optimal transfusion targets of individual blood products and to elucidate important differences in coagulation disturbance between patients by disease state and ECMO configuration. Mitigating harm from haemorrhage, thrombosis, and transfusion necessitates blood management protocols incorporating all aspects of cannulation technique, autotransfusion, and vascular and cardiothoracic surgical responses to bleeding. Restrictive transfusion policies in ECMO and reduced anticoagulation intensity regimens warrant prospective studies.

## Figures and Tables

**Table 1:** Patient demographics by ECMO type.

Variable	VA ECMO (N = 92)	VV ECMO (N = 46)	*p*-Value
Age	47.2 (16.5)	38.7 (14.7)	*p* = 0.0037
Male sex (%)	65 (70.7%)	24 (52.2%)	*p* = 0.03
Weight (kg)	81.9 (21.5)	78.1 (17.5)	*p* = 0.31
APACHE II	23 [17, 29]	22 [18, 27]	*p* = 0.88
APACHE III	80 [58, 114]	76 [61, 93]	*p* = 0.44
ICU LOS (hours)	427 [143, 661]	673 [319, 983]	*p* = 0.006
Hospital LOS (hours) Mortality (%)	664 [199, 1162] 45 (48.9%)	940 [532, 1167] 8 (17.4%)	*p* = 0.10 *p* < 0.001

APACHE = acute physiology and chronic health evaluation, LOS = length of stay.

**Table 2:** Indications for VA and VV ECMO.

Indication	VA ECMO (N = 92)	VV ECMO (N = 46)	*p*-Value
Pulmonary–ARDS	2 (2.2%)	28 (60.9%)	
Pulmonary–Other	9 (9.8%)	10 (21.7%)	
Pulmonary–Total	11 (11.9%)	38 (82.6%)	*p* < 0.001
Cardiomyopathy–MI	4 (4.3%)	0 (0.0%)	
Cardiomyopathy–Other	49 (53.3%)	0 (0.0%)	
Cardiomyopathy–Postop	23 (25.0%)	1 (2.2%)	
Cardiomyopathy–Total	76 (82.6%)	1 (2.2%)	*p* < 0.001
Sepsis	5 (5.4%)	7 (15.2%)	*p* = 0.594

ARDS = acute respiratory distress syndrome, MI = myocardial infarction.

**Table 3:** Initial Pre-ECMO status to Day 0 Trends.

VA ECMO	Pre-ECMO	Day 1	*p*-Value
Haemoglobin (g/L)	114 (26)	85 (24)	*p* < 0.001
Platelet count (×10^9^/L)	190 (99)	108 (61)	*p* < 0.001
Fibrinogen (g/L)	3.96 (2.12)	2.71 (1.83)	*p* < 0.001
**VV ECMO**			
Haemoglobin (g/L)	107 (26)	71 (34)	*p* < 0.001
Platelet count (×10^9^/L)	215 (138)	106 (124)	*p* < 0.001
Fibrinogen (g/L)	5.41 (2.61)	2.98 (1.92)	*p* < 0.001

**Table 4:** Correlation of INR and APTT in VA and VV ECMO survivors and nonsurvivors over 28 days.

Variable	Median [IQR]	β (95% CI)	*p*-Value
VV ECMO Survivor			
INR	1.1 [1.1, 1.2]	−0.015 (−0.027, −0.003)	0.015
APTT	49 [39, 63]	−1.180 (−1.553, −0.806)	<0.001
VA ECMO Survivor			
INR	1.2 [1.1, 1.5]	−0.051 (−0.081, −0.022)	0.001
APTT	49 [38, 68]	−1.752 (−2.516, −0.987)	<0.001
VV ECMO Nonsurvivor			
INR	1.1 [1.0, 1.2]	0.000 (−0.014, +0.014)	0.969
APTT	44 [36, 62]	−0.229 (−1.010, +0.552)	0.566
VA ECMO Nonsurvivor			
INR	1.3 [1.2, 1.7]	−0.051 (−0.081, −0.022)	0.001
APTT	50 [41, 74]	−2.51 (−3.43, −1.59)	<0.001

**Table 5:** Total number of transfused blood products in VA and VV ECMO patients.

Product Units	VA ECMO	VV ECMO
PRBC	2455 (3.8/ECMO day)	807 (1.0/ECMO day)
Platelets	636 (0.9/ECMO day)	125 (0.2/ECMO day)
Cryoprecipitate	1189 (1.8/ECMO day)	464 (0.6/ECMO day)

Calculated from total ECMO days for each respective modality.

## Data Availability

Available at reasonable request from the corresponding author.

## Supplementary Materials

The following supporting information can be downloaded at: https://www.mdpi.com/article/10.3390/jcm12072629/s1, Figure S1: Probability of Packed red blood cell transfusion by Fibrinogen level; Table S1: Indications by Diagnosis VVECMO; Table S2: Indications by Diagnosis VAECMO.

## References

[1] Bartlett R.H., Gattinoni L. (2010). Current status of extracorporeal life support (ECMO) for cardiopulmonary failure. Minerva Anestesiol.

[2] Peek G.J., Mugford M., Tiruvoipati R., Wilson A., Allen E., Thalanany M.M., Hibbert C.L., Truesdale A., Clemens F., Cooper N. (2009). Efficacy and economic assessment of conventional ventilatory support versus extracorporeal membrane oxygenation for severe adult respiratory failure (CESAR): A multicentre randomised controlled trial. Lancet.

[3] Allen S., Holena D., McCunn M., Kohl B., Sarani B. (2011). A review of the fundamental principles and evidence base in the use of extracorporeal membrane oxygenation (ECMO) in critically ill adult patients. J. Intensiv. Care Med..

[4] Ang A.L., Teo D., Lim C.H., Leou K.K., Tien S.L., Koh M.B. (2009). Blood transfusion requirements and independent predictors of increased transfusion requirements among adult patients on extracorporeal membrane oxygenation—A single centre experience. Vox Sang..

[5] Hein E., Munthe-Fog L., Thiara A.S., Fiane A.E., Mollnes T.E., Garred P. (2015). Heparin-coated cardiopulmonary bypass circuits selectively deplete the pattern recognition molecule ficolin-2 of the lectin complement pathway in vivo. Clin. Exp. Immunol..

[6] Ontaneda A., Annich G.M. (2018). Novel Surfaces in Extracorporeal Membrane Oxygenation Circuits. Front. Med..

[7] Lindholm L., Westerberg M., Bengtsson A., Ekroth R., Jensen E., Jeppsson A. (2004). A closed perfusion system with heparin coating and centrifugal pump improves cardiopulmonary bypass biocompatibility in elderly patients. Ann. Thorac. Surg..

[8] Morgan I.S., Codispoti M., Sanger K., Mankad P.S. (1998). Superiority of centrifugal pump over roller pump in paediatric cardiac surgery: Prospective randomised trial. Eur. J. Cardiothorac. Surg..

[9] Aubron C., Cheng A.C., Pilcher D., Leong T., Magrin G., Cooper D.J., Scheinkestel C., Pellegrino V. (2013). Factors associated with outcomes of patients on extracorporeal membrane oxygenation support: A 5-year cohort study. Crit. Care.

[10] Thiagarajan R.R., Barbaro R.P., Rycus P.T., Mcmullan D.M., Conrad S.A., Fortenberry J.D., Paden M.L. (2017). Extracorporeal Life Support Organization Registry International Report 2016. ASAIO J..

[11] Malfertheiner M.V., Philipp A., Lubnow M., Zeman F., Enger T.B., Bein T., Lunz D., Schmid C., Müller T., Lehle K. (2016). Hemostatic Changes During Extracorporeal Membrane Oxygenation: A Prospective Randomized Clinical Trial Comparing Three Different Extracorporeal Membrane Oxygenation Systems. Crit. Care Med..

[12] Murphy D.A., Hockings L.E., Andrews R.K., Aubron C., Gardiner E.E., Pellegrino V.A., Davis A.K. (2015). Extracorporeal membrane oxygenation-hemostatic complications. Transfus. Med. Rev..

[13] Figueroa Villalba C.A., Brogan T.V., McMullan D.M., Yalon L., Jordan D.I., Chandler W.L. (2020). Conversion From Activated Clotting Time to Anti-Xa Heparin Activity Assay for Heparin Monitoring During Extracorporeal Membrane Oxygenation. Crit. Care Med..

[14] Millar J.E., Fanning J.P., McDonald C.I., McAuley D.F., Fraser J.F. (2016). The inflammatory response to extracorporeal membrane oxygenation (ECMO): A review of the pathophysiology. Crit. Care.

[15] Wang S., Krawiec C., Patel S., Kunselman A.R., Song J., Lei F., Baer L.D., Ündar A. (2015). Laboratory Evaluation of Hemolysis and Systemic Inflammatory Response in Neonatal Nonpulsatile and Pulsatile Extracorporeal Life Support Systems. Artif. Organs.

[16] Hirthler M., Simoni J., Dickson M. (1992). Elevated levels of endotoxin, oxygen-derived free radicals, and cytokines during extracorporeal membrane oxygenation. J. Pediatr. Surg..

[17] Hoffman M., Monroe D.M. (2001). A cell-based model of hemostasis. Thromb. Haemost..

[18] Nair P., Hoechter D.J., Buscher H., Venkatesh K., Whittam S., Joseph J., Jansz P. (2015). Prospective observational study of hemostatic alterations during adult extracorporeal membrane oxygenation (ECMO) using point-of-care thromboelastometry and platelet aggregometry. J. Cardiothorac. Vasc. Anesth..

[19] Passmore M.R., Ki K.K., Chan C.H., Lee T., Bouquet M., Wood E.S., Raman S., Rozencwajg S., Burrell A.J., McDonald C.I. (2020). The effect of hyperoxia on inflammation and platelet responses in an ex vivo extracorporeal membrane oxygenation circuit. Artif. Organs.

[20] Orime Y., Shiono M., Hata H., Yagi S., Tsukamoto S., Okumura H., Nakata K.I., Kimura S.I., Hata M., Sezai A. (1999). Cytokine and endothelial damage in pulsatile and nonpulsatile cardiopulmonary bypass. Artif. Organs.

[21] Koning N.J., Vonk A.B., Vink H., Boer C. (2016). Side-by-Side Alterations in Glycocalyx Thickness and Perfused Microvascular Density During Acute Microcirculatory Alterations in Cardiac Surgery. Microcirculation.

[22] Fuchs G., Berg N., Broman L.M., Prahl Wittberg L. (2018). Flow-induced platelet activation in components of the extracorporeal membrane oxygenation circuit. Sci. Rep..

[23] Gross-Hardt S., Hesselmann F., Arens J., Steinseifer U., Vercaemst L., Windisch W., Brodie D., Karagiannidis C. (2019). Low-flow assessment of current ECMO/ECCO2R rotary blood pumps and the potential effect on hemocompatibility. Crit. Care.

[24] Timothy M.M., Massicotte M.P., Peter D.W., Michael S.F. (2016). ECMO Biocompatibility: Surface Coatings, Anticoagulation, and Coagulation Monitoring. Extracorporeal Membrane Oxygenation.

[25] Reynolds M.M., Annich G.M. (2011). The artificial endothelium. Organogenesis.

[26] Heilmann C., Trummer G., Beyersdorf F., Brehm K., Berchtold-Herz M., Schelling J., Geisen U., Zieger B. (2017). Acquired Von Willebrand syndrome in patients on long-term support with HeartMate II. Eur. J. Cardiothorac. Surg..

[27] Heilmann C., Geisen U., Beyersdorf F., Nakamura L., Benk C., Trummer G., Berchtold-Herz M., Schlensak C., Zieger B. (2012). Acquired von Willebrand syndrome in patients with extracorporeal life support (ECLS). Intensive Care Med..

[28] Vamvakas E.C., Blajchman M.A. (2007). Transfusion-related immunomodulation (TRIM): An update. Blood Rev..

[29] Cuschieri J., Freeman B., O’Keefe G., Harbrecht B.G., Bankey P., Johnson J.L., Minei J.P., Sperry J., West M.A., Nathens A. (2008). Inflammation and the host response to injury a large-scale collaborative project: Patient-oriented research core standard operating procedure for clinical care X. Guidelines for venous thromboembolism prophylaxis in the trauma patient. J. Trauma.

[30] Smith A., Hardison D., Bridges B., Pietsch J. (2013). Red blood cell transfusion volume and mortality among patients receiving extracorporeal membrane oxygenation. Perfusion.

[31] Martucci G., Panarello G., Occhipinti G., Ferrazza V., Tuzzolino F., Bellavia D., Sanfilippo F., Santonocito C., Bertani A., Vitulo P. (2019). Anticoagulation and Transfusions Management in Veno-Venous Extracorporeal Membrane Oxygenation for Acute Respiratory Distress Syndrome: Assessment of Factors Associated With Transfusion Requirements and Mortality. J. Intensiv. Care Med..

[32] Hodgson C.L.F.B., Salimi F., Anderson S., Bernard S., Board J.V., Brodie D., Buhr H., Burrell A.J.C., Cooper D.J., Fan E. (2022). The EXCEL Registry Report 2019–2021.

[33] ELSO (2022). ECLS Registry Report-International Summary October 2022 [Online Registry]. https://www.elso.org/portals/0/files/reports/2022_october/international%20report%20october.pdf.

[34] Elsharkawy H.A., Li L., Esa W.A., Sessler D.I., Bashour C.A. (2010). Outcome in patients who require venoarterial extracorporeal membrane oxygenation support after cardiac surgery. J. Cardiothorac. Vasc. Anesth..

[35] Rastan A.J., Dege A., Mohr M., Doll N., Falk V., Walther T., Mohr F.W. (2010). Early and late outcomes of 517 consecutive adult patients treated with extracorporeal membrane oxygenation for refractory postcardiotomy cardiogenic shock. J. Thorac. Cardiovasc. Surg..

[36] Loungani R.S., Fudim M., Ranney D., Kochar A., Samsky M.D., Bonadonna D., Itoh A., Takayama H., Takeda K., Wojdyla D. (2021). Contemporary Use of Venoarterial Extracorporeal Membrane Oxygenation: Insights from the Multicenter RESCUE Registry. J. Card. Fail..

[37] Hughes T., Zhang D., Nair P., Buscher H. (2021). A Systematic Literature Review of Packed Red Cell Transfusion Usage in Adult Extracorporeal Membrane Oxygenation. Membranes.

[38] Lotz C., Streiber N., Roewer N., Lepper P.M., Muellenbach R.M., Kredel M. (2017). Therapeutic Interventions and Risk Factors of Bleeding During Extracorporeal Membrane Oxygenation. ASAIO J..

[39] Mazzeffi M., Greenwood J., Tanaka K., Menaker J., Rector R., Herr D., Kon Z., Lee J., Griffith B., Rajagopal K. (2016). Bleeding, Transfusion, and Mortality on Extracorporeal Life Support: ECLS Working Group on Thrombosis and Hemostasis. Ann. Thorac. Surg..

[40] Agerstrand C.L., Burkart K.M., Abrams D.C., Bacchetta M.D., Brodie D. (2015). Blood conservation in extracorporeal membrane oxygenation for acute respiratory distress syndrome. Ann. Thorac. Surg..

[41] Ferraris V.A., Davenport D.L., Saha S.P., Bernard A., Austin P.C., Zwischenberger J.B. (2011). Intraoperative transfusion of small amounts of blood heralds worse postoperative outcome in patients having noncardiac thoracic operations. Ann. Thorac. Surg..

[42] ELSO (2017). Extracorporeal Life Support Organization Guidelines for Adult Respiratory Failure. https://www.elso.org/Portals/0/ELSO%20Guidelines%20For%20Adult%20Respiratory%20Failure%201_4.pdf.

[43] Sawyer A.A., Wise L., Ghosh S., Bhatia J., Stansfield B.K. (2017). Comparison of transfusion thresholds during neonatal extracorporeal membrane oxygenation. Transfusion.

[44] Singh G., Nahirniak S., Arora R., Légaré J.F., Kanji H.D., Nagpal D., Lamarche Y., Fan E., Parhar K.K.S. (2020). Transfusion Thresholds for Adult Respiratory Extracorporeal Life Support: An Expert Consensus Document. Can. J. Cardiol..

[45] Martucci G., Grasselli G., Tanaka K., Tuzzolino F., Panarello G., Schmidt M., Bellani G., Arcadipane A. (2019). Hemoglobin trigger and approach to red blood cell transfusions during veno-venous extracorporeal membrane oxygenation: The international TRAIN-ECMO survey. Perfusion.

[46] Ducrocq G., Gonzalez-Juanatey J.R., Puymirat E., Lemesle G., Cachanado M., Durand-Zaleski I., Arnaiz J.A., Martínez-Sellés M., Silvain J., Ariza-Solé A. (2021). Effect of a Restrictive vs Liberal Blood Transfusion Strategy on Major Cardiovascular Events Among Patients With Acute Myocardial Infarction and Anemia: The REALITY Randomized Clinical Trial. JAMA.

[47] Carson J.L., Stanworth S.J., Dennis J.A., Trivella M., Roubinian N., Fergusson D.A., Triulzi D., Dorée C., Hébert P.C. (2021). Transfusion thresholds for guiding red blood cell transfusion. Cochrane Database Syst. Rev..

[48] Holst L.B., Haase N., Wetterslev J., Wernerman J., Guttormsen A.B., Karlsson S., Johansson P.I., Åneman A., Vang M.L., Winding R. (2014). Lower versus higher hemoglobin threshold for transfusion in septic shock. N. Engl. J. Med..

[49] Kim H.S., Park S. (2017). Blood Transfusion Strategies in Patients Undergoing Extracorporeal Membrane Oxygenation. Korean J. Crit. Care Med..

[50] Hébert P.C., Wells G., Blajchman M.A., Marshall J., Martin C., Pagliarello G., Tweeddale M., Schweitzer I., Yetisir E., Transfusion Requirements in Critical Care Investigators for the Canadian Critical Care Trials Group (1999). A multicenter, randomized, controlled clinical trial of transfusion requirements in critical care. Transfusion Requirements in Critical Care Investigators, Canadian Critical Care Trials Group. N. Engl. J. Med..

[51] Fiser R.T., Irby K., Ward R.M., Tang X., McKamie W., Prodhan P., Corwin H.L. (2014). RBC Transfusion in Pediatric Patients Supported with Extracorporeal Membrane Oxygenation: Is There an Impact on Tissue Oxygenation?. Pediatr. Crit. Care Med..

[52] Abbasciano R.G., Yusuff H., Vlaar A.P.J., Lai F., Murphy G.J. (2021). Blood Transfusion Threshold in Patients Receiving Extracorporeal Membrane Oxygenation Support for Cardiac and Respiratory Failure-A Systematic Review and Meta-Analysis. J. Cardiothorac. Vasc. Anesth..

[53] Ramanathan K., MacLaren G., Combes A., Brodie D., Shekar K. (2020). Blood transfusion strategies and ECMO during the COVID-19 pandemic-Authors’ reply. Lancet Respir. Med..

[54] Bembea M.M., Annich G., Rycus P., Oldenburg G., Berkowitz I., Pronovost P. (2013). Variability in anticoagulation management of patients on extracorporeal membrane oxygenation: An international survey. Pediatr. Crit. Care Med..

[55] Doyle A.J., Richardson C., Sanderson B., Wong K., Wyncoll D., Camporota L., Barrett N.A., Hunt B.J., Retter A. (2020). Restrictive Transfusion Practice in Adults Receiving Venovenous Extracorporeal Membrane Oxygenation: A Single-Center Experience. Crit. Care Explor..

[56] Martucci G., Schmidt M., Agerstrand C., Tabatabai A., Tuzzolino F., Giani M., Ramanan R., Grasselli G., Schellongowski P., Riera J. (2022). Transfusion practice in patients receiving VV ECMO (PROTECMO): A prospective, multicentre, observational study. Lancet Respir. Med..

[57] Swol J., Marschall C., Strauch J.T., Schildhauer T.A. (2018). Hematocrit and impact of transfusion in patients receiving extracorporeal life support. Perfusion.

[58] Cahill C.M., Blumberg N., Schmidt A.E., Knight P.A., Melvin A.L., Massey H.T., Delehanty J.M., Zebrak S.B., Refaai M.A. (2018). Implementation of a Standardized Transfusion Protocol for Cardiac Patients Treated With Venoarterial Extracorporeal Membrane Oxygenation Is Associated With Decreased Blood Component Utilization and May Improve Clinical Outcome. Anesth. Analg..

[59] Tolksdorf B., Schmeck J., Osika A., Bender H.J., Quintel M. (2000). Autotransfusion during extracorporeal membrane oxygenation. Int. J. Artif. Organs.

[60] Mueller M.M., Van Remoortel H., Meybohm P., Aranko K., Aubron C., Burger R., Carson J.L., Cichutek K., De Buck E., Devine D. (2019). Patient Blood Management: Recommendations From the 2018 Frankfurt Consensus Conference. JAMA.

[61] Danial P., Hajage D., Nguyen L.S., Mastroianni C., Demondion P., Schmidt M., Bouglé A., Amour J., Leprince P., Combes A. (2018). Percutaneous versus surgical femoro-femoral veno-arterial ECMO: A propensity score matched study. Intensiv. Care Med..

[62] Maggio P., Hemmila M., Haft J., Bartlett R. (2007). Extracorporeal life support for massive pulmonary embolism. J. Trauma.

[63] Subramaniam A.V., Barsness G.W., Vallabhajosyula S., Vallabhajosyula S. (2019). Complications of Temporary Percutaneous Mechanical Circulatory Support for Cardiogenic Shock: An Appraisal of Contemporary Literature. Cardiol. Ther..

[64] Ostermann M., Lumlertgul N. (2021). Acute kidney injury in ECMO patients. Crit. Care.

[65] Thongprayoon C., Cheungpasitporn W., Lertjitbanjong P., Aeddula N.R., Bathini T., Watthanasuntorn K., Srivali N., Mao M.A., Kashani K. (2019). Incidence and Impact of Acute Kidney Injury in Patients Receiving Extracorporeal Membrane Oxygenation: A Meta-Analysis. J. Clin. Med..

[66] Ostermann M., Connor M., Kashani K. (2018). Continuous renal replacement therapy during extracorporeal membrane oxygenation: Why, when and how?. Curr. Opin. Crit. Care.

[67] Grandin E.W., Nunez J.I., Willar B., Kennedy K., Rycus P., Tonna J.E., Kapur N.K., Shaefi S., Garan A.R. (2022). Mechanical Left Ventricular Unloading in Patients Undergoing Venoarterial Extracorporeal Membrane Oxygenation. J. Am. Coll. Cardiol..

[68] Lorusso R., Meani P., Raffa G.M., Kowalewski M. (2022). Extracorporeal membrane oxygenation and left ventricular unloading: What is the evidence?. JTCVS Tech..

[69] Kaseer H., Soto-Arenall M., Sanghavi D., Moss J., Ratzlaff R., Pham S., Guru P. (2020). Heparin vs bivalirudin anticoagulation for extracorporeal membrane oxygenation. J. Card. Surg..

[70] Lind S.E., Boyle M.E., Fisher S., Ishimoto J., Trujillo T.C., Kiser T.H. (2014). Comparison of the aPTT with alternative tests for monitoring direct thrombin inhibitors in patient samples. Am. J. Clin. Pathol..

[71] Mazzeffi M.A., Tanaka K., Roberts A., Rector R., Menaker J., Kon Z., Deatrick K.B., Kaczorowski D., Griffith B., Herr D. (2019). Bleeding, Thrombosis, and Transfusion With Two Heparin Anticoagulation Protocols in Venoarterial ECMO Patients. J. Cardiothorac. Vasc. Anesth..

[72] Rhoades R., Leong R., Kopenitz J., Thoma B., McDermott L., Dovidio J., Barletti S., Gong J.Z., Massey H.T., McKenzie S.E. (2021). Coagulopathy monitoring and anticoagulation management in COVID-19 patients on ECMO: Advantages of a heparin anti-Xa-based titration strategy. Thromb. Res..

[73] Irby K., Swearingen C., Byrnes J., Bryant J., Prodhan P., Fiser R. (2014). Unfractionated heparin activity measured by anti-factor Xa levels is associated with the need for extracorporeal membrane oxygenation circuit/membrane oxygenator change: A retrospective pediatric study. Pediatr. Crit. Care Med..

[74] Chlebowski M.M., Baltagi S., Carlson M., Levy J.H., Spinella P.C. (2020). Clinical controversies in anticoagulation monitoring and antithrombin supplementation for ECMO. Crit. Care.

[75] Niimi K.S., Fanning J.J. (2014). Initial experience with recombinant antithrombin to treat antithrombin deficiency in patients on extracorporeal membrane oxygenation. J. Extra Corpor. Technol..

[76] Byrnes J.W., Swearingen C.J., Prodhan P., Fiser R., Dyamenahalli U. (2014). Antithrombin III supplementation on extracorporeal membrane oxygenation: Impact on heparin dose and circuit life. ASAIO J..

[77] Gorbet M.B., Sefton M.V. (2004). Biomaterial-associated thrombosis: Roles of coagulation factors, complement, platelets and leukocytes. Biomaterials.

[78] Cartwright B., Bruce H.M., Kershaw G., Cai N., Othman J., Gattas D., Robson J.L., Hayes S., Alicajic H., Hines A. (2021). Hemostasis, coagulation and thrombin in venoarterial and venovenous extracorporeal membrane oxygenation: The HECTIC study. Sci. Rep..

[79] Loscalzo J. (2012). From clinical observation to mechanism--Heyde’s syndrome. N. Engl. J. Med..

[80] Wu Y. (2015). Contact pathway of coagulation and inflammation. Thromb. J..

[81] Schneider S.W., Nuschele S., Wixforth A., Gorzelanny C., Alexander-Katz A., Netz R.R., Schneider M.F. (2007). Shear-induced unfolding triggers adhesion of von Willebrand factor fibers. Proc. Natl. Acad. Sci. USA.

[82] Heilmann C., Geisen U., Beyersdorf F., Nakamura L., Benk C., Berchtold-Herz M., Trummer G., Schlensak C., Zieger B. (2010). Acquired von Willebrand syndrome in patients with ventricular assist device or total artificial heart. Thromb. Haemost..

[83] Ki K.K., Passmore M.R., Chan C.H., Malfertheiner M.V., Fanning J.P., Bouquet M., Millar J.E., Fraser J.F., Suen J.Y. (2019). Low flow rate alters haemostatic parameters in an ex-vivo extracorporeal membrane oxygenation circuit. Intensiv. Care Med. Exp..

[84] Wu J., Paden B.E., Borovetz H.S., Antaki J.F. (2010). Computational fluid dynamics analysis of blade tip clearances on hemodynamic performance and blood damage in a centrifugal ventricular assist device. Artif. Organs.

[85] The Australia and New Zealand Extracorporeal Membrane Oxygenation (ANZ ECMO) Influenza Investigators (2009). Extracorporeal Membrane Oxygenation for 2009 Influenza A(H1N1) Acute Respiratory Distress Syndrome. JAMA.

[86] Aubron C., McQuilten Z., Bailey M., Board J., Buhr H., Cartwright B., Dennis M., Hodgson C., Forrest P., McIlroy D. (2019). Low-Dose Versus Therapeutic Anticoagulation in Patients on Extracorporeal Membrane Oxygenation: A Pilot Randomized Trial. Crit. Care Med..

[87] Alkazemi A., Eche I.M., Adra M., Cabezas F., Patel P., Rick K., Grandin E.W. (2022). Conventional versus restricted anti-Xa-guided heparin protocol in adult patients undergoing extracorporeal membrane oxygenation. Artif. Organs.

[88] Muellenbach R.M., Kredel M., Kunze E., Kranke P., Kuestermann J., Brack A., Gorski A., Wunder C., Roewer N., Wurmb T. (2012). Prolonged heparin-free extracorporeal membrane oxygenation in multiple injured acute respiratory distress syndrome patients with traumatic brain injury. J. Trauma Acute Care Surg..

