# Supplementary material for: Haematological Trends and Transfusion during Adult Extracorporeal Membrane Oxygenation: A Single Centre Study
Source: J Clin Med. 2023 Mar 31;12(7):2629. doi: 10.3390/jcm12072629 (PMC10095131; doi:10.3390/jcm12072629)
Supplement: Supplementary file 1 [file jcm-12-02629-s001.zip › jcm-2245057-supplementary.pdf]

Supplementary Table S1:
Indications by Diagnosis VVECMO

		VVECMO (n=46)
ARDS (n=28)	Influenza A Virus	15
	Non Influenza Respiratory Viruses	5
	Bacterial ARDS	8
Non ARDS (n=18)	Post-Lung transplant	8
	Bridge to Lung Transplant, Pulmonary haemorrhage	2
	Sepsis/Cardiomyopathy	7
	Postoperative cardiomyopathy	1

Supplementary Table S2:
Indications by Diagnosis VAECMO

		VAECMO (n=92)
Post Cardiotomy (n=23)	Cardiac Surgery (non-transplant)	21
	Post Heart Transplant	2
Non Cardiotomy (n=69)		
	AMI Cardiogenic Shock	4
	ECPR	11
	Dilated Cardiomyopathy	8
	Post-partum cardiomyopathy	1
	Giant Cell myocarditis	3
	Myocarditis (unknown aetiology)	10
	Post lung-transplant cardiomyopathy	8
	Unspecified Cardiomyopathy	8
	ARDS	2
	Pulmonary other	9
	Sepsis	5

Supplementary Figure S1:
Probability of Packed red blood cell transfusion by Fibrinogen level

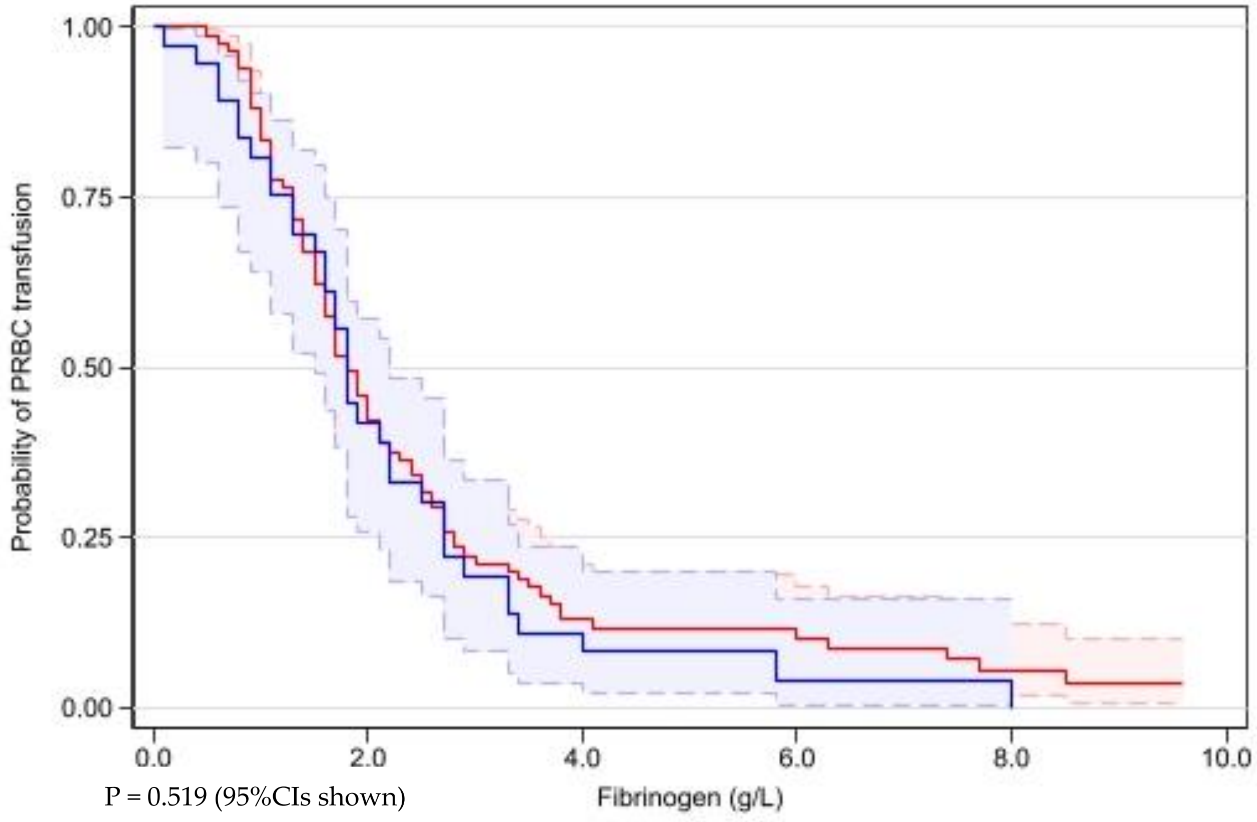

P = 0.519 (95%CIs shown)
VA= red, VV= blue
